# Characterization of a novel *HDAC2* pathogenetic variant: a missing puzzle piece for chromatinopathies

**DOI:** 10.1007/s00439-024-02675-0

**Published:** 2024-05-16

**Authors:** Elisabetta Di Fede, Antonella Lettieri, Esi Taci, Silvia Castiglioni, Stefano Rebellato, Chiara Parodi, Elisa Adele Colombo, Paolo Grazioli, Federica Natacci, Paola Marchisio, Lidia Pezzani, Grazia Fazio, Donatella Milani, Valentina Massa, Cristina Gervasini

**Affiliations:** 1 Department of Health Sciences, Università degli Studi di Milano, Milan, Italy; 2 “Aldo Ravelli” Center for Neurotechnology and Experimental Brain Therapeutics, Università degli Studi di Milano, Milan, Italy; 3 Tettamanti Center, Fondazione IRCCS San Gerardo dei Tintori, Monza, Italy; 4 School of Medicine and Surgery, University of Milano-Bicocca, Monza, 20900 Italy; 5 Fondazione IRCCS Ca’ Granda Ospedale Maggiore Policlinico di Milano, Milan, Italy; 6 Department of Pathophysiology and Transplantation, Università degli Studi di Milano, Milan, Italy

## Abstract

Histone deacetylases (HDACs) are enzymes pivotal for histone modification (i.e. acetylation marks removal), chromatin accessibility and gene expression regulation. Class I HDACs (including HDAC1, 2, 3, 8) are ubiquitously expressed and they often participate in multi-molecular protein complexes. To date, three neurodevelopmental disorders caused by mutations in genes encoding for HDACs (*HDAC4*, *HDAC6* and *HDAC8*) and thus belonging to the group of chromatinopathies, have been described. We performed whole exome sequencing (WES) for a patient (#249) clinically diagnosed with the chromatinopathy Rubinstein-Taybi syndrome (RSTS) but negative for mutations in RSTS genes, identifying a *de novo* frameshift variant in *HDAC2* gene. We then investigated its molecular effects in lymphoblastoid cell lines (LCLs) derived from the patient compared to LCLs from healthy donors (HD). As the variant was predicted to be likely pathogenetic and to affect the sequence of nuclear localization signal, we performed immunocytochemistry and lysates fractionation, observing a nuclear mis-localization of HDAC2 compared to HD LCLs. In addition, HDAC2 total protein abundance resulted altered in patient, and we found that newly identified variant in *HDAC2* affects also acetylation levels, with significant difference in acetylation pattern among patient #249, HD and RSTS cells and in expression of a known molecular target. Remarkably, RNA-seq performed on #249, HD and RSTS cells shows differentially expressed genes (DEGs) common to #249 and RSTS. Interestingly, our reported patient was clinically diagnosed with RSTS, a chromatinopathy which known causative genes encode for enzymes antagonizing HDACs. These results support the role of *HDAC2* as causative gene for chromatinopathies, strengthening the genotype-phenotype correlations in this relevant group of disorders.

## Introduction

Epigenetic modifications are fundamental for chromatin accessibility and its control in gene expression regulation in several biological processes, in particular in development and differentiation (Atlasi and Stunnenberg 2017). Opened or closed chromatin status is associated with active or repressed transcriptional programs, respectively, and it is dynamically determined by different epigenetic marks. Among these, acetylation on histone tails of specific lysine residues contributes to transcriptional activation thanks to enzymes known as histone acetyltransferases (HATs), while histone deacetylases (HDACs) are responsible for acetylated marks removal, thus antagonizing HATs functions. HDACs are divided into four classes based on their structure similarities and catalytic mechanisms: Class I (HDAC1, 2, 3, 8), Class IIa (HDAC4, 5, 7, 9), Class IIb (HDAC6, 10), Class III (SIRT1-7) and Class IV (HDAC11). Class I, II and IV enzymes require zinc for catalysis, while sirtuin proteins (Class III) use nicotinamide adenine dinucleotide (NAD^+^) as a cofactor (Ali et al. 2018). Class I HDACs are expressed ubiquitously, they are mainly localized into the nucleus and often found in multi-molecular protein complexes - e.g. HDAC1 and HDAC2 are components of Sin3, NuRD and CoREST nuclear coregulatory complexes (You et al. 2001). Class IIa HDACs have an expression pattern which varies among family members, ranging from brain and heart to muscles and skeleton (Chang et al. 2004; Vega et al. 2004), and these enzymes can carry out their repressor activity by either recruiting class I HDACs or interacting with other transcriptional factors. Among class IIb and IV HDACs, little is known so far about HDAC10 and HDAC11, while HDAC6 is the main tubulin deacetylase implicated in cytoskeleton regulation (Zhang et al. 2008).

To date, three neurodevelopmental disorders caused by mutations in genes encoding for HDACs are recognized. Mutations in *HDAC4* gene are causative of Neurodevelopmental disorder with central hypotonia and dysmorphic facies (OMIM #619797). These patients display developmental and intellectual delay, seizures, distinctive facial features, delayed closure of the anterior fontanel, nonspecific brain abnormalities and scoliosis (Wakeling et al. 2021), although variable phenotypes have been reported among individuals with *HDAC4* haploinsufficiency (Villavicencio-Lorini et al. 2013; Wheeler et al. 2014; Jean-Marçais et al. 2015; Sun and Wan 2016; Squeo et al. 2020). *HDAC6* has been reported as causative gene of Chondrodysplasia with platyspondyly, distinctive brachydactyly, hydrocephaly and microphthalmia (OMIM #300863), a syndrome characterized by skeletal anomalies, macrocephaly, facial dysmorphisms, developmental delay and cognitive impairment, with milder phenotypes observed in females (Simon et al. 2010). Finally, pathogenetic variants in *HDAC8* are causative of Cornelia de Lange syndrome 5 (CdLS5, OMIM #300882), whose patients show growth impairment, ID, multi-organ anomalies and peculiar facial features (Deardorff et al. 2012a; Kaiser et al. 2014; Parenti et al. 2016). Due to HDACs function, these syndromes belong to the group of chromatinopathies, rare genetic disorders caused by mutations in genes of the epigenetic machinery (Fahrner and Bjornsson 2019).

Here we report a patient with initial diagnosis of Rubinstein-Taybi syndrome (RSTS, OMIM #180849, #613684), another chromatinopathy which causative genes *CREBBP* or *EP300* encode for two HATs (Petrif et al. 1995; Roelfsema et al. 2005). Since this patient resulted negative for mutations in RSTS genes, she underwent whole exome sequencing (WES) and was found to be carrier of a *de novo* pathogenetic frameshift variant in *HDAC2*. After its validation, we investigated the molecular effects of the novel mutation in lymphoblastoid cell line (LCLs). In particular, we assessed in patient LCL HDAC2 intracellular localization, its expression and protein abundance, and eventually downstream effects of the variant on acetylation pattern and gene expression, which was investigated also by transcriptomic analysis.

## Materials and methods

### Subject

Patient #249 was clinically and phenotypically evaluated by an expert clinical geneticist (DM) who proposed a clinical diagnosis of RSTS. The subject and its family gave informed consent.

### Exome sequencing and variant validation

Genomic DNA of patient (#249) and its parents was extracted from blood and saliva samples by Wizard Genomic DNA Purification Kit (A1120, Promega) and Quick-DNA Miniprep Plus Kit (D4068, Zymo Research) respectively. DNA obtained from whole blood was used for library preparation and exome enrichment with Agilent SureSelect V7 kit according to manufacturer protocol and an indexed 150 bp paired-end sequencing was carried out on Illumina HiSeq3000 instrument at CRS4 NGS facility. Data were analyzed exploiting an analysis pipeline based on public tools and reported in Di Fede et al. 2020 (Di Fede et al. 2020).

Extracted blood and saliva DNA of the family trio was used for PCR reactions according to the protocol of GoTaq Flexi DNA Polymerase (M8296, Promega). After PCR amplification, Sanger sequencing was performed with the same pair of primers to confirm the variant of interest identified through exome sequencing, using ENST00000519065.6 (NM_001527.4) as reference for *HDAC2*. In addition, RNA of patient was extracted isolating PBMC from whole blood by Ficoll-Paque™ PLUS (#11,768,538, Cytiva-Danaher Corporation) and performing the RNA extraction according to TRI Reagent® protocol (#93,289, Merck KGaA). RNA was retrotranscribed using SensiFAST™ cDNA Synthesis Kit (BIO-65,054, Meridian Bioscience) according to manufacturer’s protocol and analysis of the transcript was carried out by RT-PCR followed by Sanger sequencing. HGVS nomenclature guidelines were observed for sequence variant which was reported as individual ID #00442016 in the LOVD website (https://databases.lovd.nl/shared/individuals/00442016).

### Cell culture

Patient #249, three RSTS patients with *CREBBP* mutations (*CREBBP*^*mut*^) and six healthy donors (HD) lymphoblastoid cell lines (LCLs) were obtained in synergy with Telethon Network of Genetic Biobanks (Gaslini Genetic Bank, Genova, IT) (Table 1) (Lopez-Atalaya et al. 2012; Spena et al. 2015; Baldo et al. 2016). Ethics Committee of Università degli Studi di Milano approved the use of these cell lines (Comitato Etico number 99/20, 17 November 2020). Cells were cultured in RPMI 1640 medium supplemented with L-glutamine (L0498-500, Aurogene s.r.l), 20% fetal bovine serum (ECS0180L, Euroclone), and penicillin/streptomycin (ECB3001, Euroclone), with 5% CO_2_ and at 37 °C.

**Table 1:** Patients-derived LCLs used in the present work

Gene	LCLs	cDNA change	Protein change	Mutation type	Reference
***HDAC2***	#249	c.1330_1333del	p.(K444Lfs*61)	Frameshift	this study
***CREBBP***	RSTS 114	c.4485-7G > C	p.(R1428_G1465del) p.(F1379_G1465del)	Splicing	Lopez-Atalaya et al. 2012
	RSTS 122	c.4394 + 5G > T	p.(R1428_G1465del) p.(F1379_G1465del)	Splicing	Spena et al. 2015
	RSTS 176	c.4508 A > T	p.(Y1503F)	Missense (KAT)	Spena et al. 2015

### Immunocytochemistry

At least 1.5 × 10^4^ cultured LCLs were seeded in duplicate on SuperFrost Plus slides (#10,149,870, Thermofisher Scientific) through cytospin for 5 min at 500 rpm. Slides were incubated with PFA 4% for 10 min and washed three times with PBS (Phosphate-Buffered Saline). In a wet chamber cells fixed on slides were firstly permeabilized with PBT (PBS with 0,2% Triton) for 10 min at RT and then incubated with a blocking solution (PBT supplemented with 10% FBS) for 30 min at RT. Incubation with anti-HDAC2 antibody (1:500, ab137364 Abcam) was performed overnight at 4°C. Slides were washed with PBT, incubated for 1 h and 30 min with Alexa-488 anti-Rabbit secondary antibody (1:250, #6441-30 SouthernBiotech), washed again in PBT and mounted with Everbrite Mounting Medium with DAPI (#23,002, Biotium). For each sample, about ten images of randomly selected fields were acquired by confocal microscopy A1/A1R (Nikon Corporation) at 60x magnification and an average number of 10^2^ cells was counted for each cell line. Images were blinded-counted and analyzed with ImageJ software (National Institute of Health) by three different operators. Five degrees of HDAC2 localization were set (from 0 to 4, where 0 means HDAC2 signal is absent while 4 is strongly present in the nucleus) and number of cells assigned to each degree (HDAC2 + cells) was normalized on the total cell number per image acquired.

### Protein extraction and fractionation

Cellular pellets were washed two times in PBS and centrifuged 5 min 2,500-3,000 rpm at 4 °C, resuspended in cold S300 buffer (50 mM HEPES pH 7.6, 300 mM NaCl, 0.1% NP40, 2 mM MgCl_2_, 10% glycerol) supplemented with protease inhibitors cocktail (P8340, Sigma-Aldrich) and nuclease (SC-202,391, Santa-Cruz Biotechnology) and left on ice for 1 h. Samples were centrifuged at maximum speed (13,000 rpm) for 10 min at 4 °C and the supernatant was quantified with Bradford assay (Bio-Rad) following the manufacturer’s instructions. Protein samples were finally denatured in Laemmli sample buffer 4x (LSB, #1,610,747, Bio-Rad) supplemented with β-mercaptoethanol and boiled for 10 min at 100 °C before they could be loaded on gel.

For fractionation LCLs cellular pellets were split equally in two recipients: for total lysate (Total) and cytoplasm/nuclei fraction. Pellet for total lysate was resuspended in S300 buffer for protein extraction for 30 min on ice. Pellet for cytoplasm/nuclei fraction was washed in cold hypotonic buffer (10 mM Tris HCl pH 7.6, 1.5 mM MgCl_2_, 10 mM KCl, 340 mM sucrose), centrifuged 5 min at 2,500 rpm at 4 °C, resuspended in hypotonic buffer (1:5) and put on ice for 10–15 min. Then 1/30 of Triton X100 10% was rapidly added to the solution which was left on ice for 5 min and centrifuged at maximum speed for one minute at 4 °C. At this point supernatant was collected for cytoplasmic fraction (Citosol), while cellular pellet corresponding to nuclei fraction was washed again in hypotonic buffer, lysed in S300 buffer added with protease inhibitors cocktail and nuclease, and left resting on ice for 30 min. Nuclei and total lysate were extracted as reported below and centrifuged at maximum speed for 10 min at 4 °C for removing debris. After quantification LSB with β-mercaptoethanol (#1610710 Bio-Rad) was added to fractions. Fractionation experiments were performed in triplicates with three different pairs (#249 and HD) of LCLs samples.

### Western blot

Denatured protein samples were separated by SDS-PAGE (Running buffer 1x diluted from 10x made of 3% Tris HCl, 14,4% Glycine and 1% SDS), transferred to nitrocellulose membranes in Transfer buffer 1 × (20% methanol and 10% Transfer buffer 10x, composed of 3% Tris HCl and 14,4% Glycine). Membranes were washed with TBS (TBS 1x diluted from 10x made of 3% Tris HCl, 8,7% NaCl and 0,2% KCl) supplemented with 0.1% Tween (TBS-T), blocked for 1 h at RT with 5% milk in TBS-T and then with primary antibody diluted in blocking solution for 1 h at RT (rabbit anti-H3, 1:10000, ab1791 Abcam, RRID: AB_302613; rabbit anti-GAPDH, 1:2000, #5174 Cell Signaling Technology, RRID: AB_10622025) or overnight at 4 °C (rabbit anti-HDAC2, 1:1000, ab137364 Abcam, RRID: AB_3073990; rabbit anti-HSP90, 1:1000, #4874 Cell Signaling Technology, RRID: AB_2121214; mouse anti-p53, 1:500, #MA5-12557 Thermo Fisher Scientific, RRID: AB_10989883). After washing, membranes were incubated with goat anti-rabbit or anti-mouse IgG horseradish peroxidase (HRP)-conjugated antibody (#1706515 or 1706516, Bio-Rad, RRID: AB_11125142 or RRID: AB_2921252) diluted in 5% milk in TBS-T for 1 h at RT, they were washed again and chemiluminescence signals were detected through ECL (#1705061 Bio-Rad) incubation and captured by Chemidoc Imaging System. Data obtained from western blot, performed in seven biological and a minimum of two technical replicates, were analyzed by Image Lab Software (Bio-Rad) and expressed as ratio between values from protein of interest (HDAC2, H3ac or p53) and reference protein (GAPDH or H3). Fractionation ratios were calculated comparing nuclear or cytoplasmatic fraction to total lysates.

### RNA extraction and gene expression analysis

RNA extraction from #249 and five HD LCLs pellets was obtained following TRIzol reagent protocol (#15596026 Invitrogen). Briefly, 1mL of TRIzol was added to each pellet for nucleoprotein complexes dissociation. Total RNA was purified and precipitated with chloroform and isopropanol respectively, and RNA pellets were washed two times with 75% ethanol. Once dried, pellets were resuspended in nuclease free water and RNA quantified by Nanodrop. Reverse transcription (RT) was performed using All-In-One 5X RT MasterMix (G592 Applied Biological Materials Inc., abm) according to manufacturer’s protocol, in order to retrotranscribe 1µg of RNA per sample into cDNA. RT reactions were diluted with H_2_O and quantitative real time PCR (RT-qPCR) was carried out using TB Green Premix Ex Taq (Tli RNase H Plus) (#RR420A, Takara Bio Inc.) and the CFX Opus 96 Real-Time PCR System (Bio-Rad). Experiments were assayed in biological and technical triplicates and *HDAC2* or *TP53* levels were quantified relatively to the expression of three housekeeping genes (*GAPDH*, *RPLP0*, *RPL13A*) using a comparative Ct quantification method. The following primers were used for RT-qPCR: *HDAC2* 5’-AGGCAAATACTATGCTGTC-3’ and 5’-TGAAACAACCCAGTCTATC-3’; *TP53* 5’-CTATGAGCCGAGGTTG-3’ and 5’-AGAGGAGCTGTGTTGG-3’; *GAPDH* 5’-AGCCACATCGCTCAGACAC-3’ and 5’-GCCCAATACGACCAAATCC-3’; *RPLP0* 5’-TCTACAACCCTGAAGTGCTTGAT-3’ and 5’-CAATCTGCAGACAGACACTGG-3’; *RPL13A* 5’-CCTGGAGGAGAAGAGGAAAGAGA-3’ and 5’-TTGAGGACCTCTGTGTATTTGTCAA-3’.

### AlphaLISA® assay

An amount of 1 × 10^4^ cells/well from #249, three *CREBBP*^*mut*^, and five HD LCLs was resuspended in 60 µl of RPMI for AlphaLISA assay (PerkinElmer) (Di Fede et al. 2021). Acetylation assessment was carried out according to AlphaLISA Cellular Detection Kit protocols for Acetylated-Histone H3 Lysine 27 (H3K27ac) (AL720, PerkinElmer) and unmodified Histone H3 Lysine 4 (H3K4) (AL719, PerkinElmer), used for normalization. Briefly, after incubation of biological triplicates with Cell-Histone Lysis buffer and Cell-Histone Extraction buffer for 15 and 10 min respectively, technical triplicates were incubated with Acceptor mix for 1 h at room temperature (RT), followed by addition of Donor mix and incubation overnight at RT. Signal was detected at PerkinElmer Ensight™ plate reader.

### RNA-sequencing

RNA previously extracted from #249, one RSTS and two HD LCLs were used for whole-transcriptome RNA-sequencing (RNA-Seq) analysis performed by next-generation sequencing, using the Universal RNA-Seq kit (Tecan) with targeted transcript depletion with AnyDeplete (Tecan) for ribosomal and globin genes. The yields of final libraries were assessed by Qubit 4.0 fluorimeter, and their sizes were measured by Agilent Bioanalyzer. The libraries were analyzed by paired-end sequencing on NextSeq2000 Illumina platform, 2 × 50. FASTQ files are available in the BioStudies-ArrayExpress database (https://www.ebi.ac.uk/biostudies/ArrayExpress/studies) under accession number E-MTAB-14,042. Raw FASTQ sequences were quality-tested with FastQC (https://www.bioinformatics.babraham.ac.uk/projects/fastqc/) and aligned against the GRCh38/hg38 reference human genome with the splice-aware aligner STAR v2.7.10b (Dobin et al. 2013). Sorted, indexed BAM alignment files were used for quantification with featureCounts (v2.0.0) (Liao et al. 2014) considering only uniquely mapped reads. GRCh38 Ensembl Release 108 annotation was used as reference. The Bioconductor package DESeq2 v1.30 (Love et al. 2014) was used to perform differential gene expression analysis in addition to custom shell and R scripts. Significant gene sets were selected based on nominal p value less than 0.05 and absolute log2(0.58) which translates to an actual foldchange higher than 1.5 or lower than 0.67.

### Statistical analysis

Data were analyzed using Prism software (GraphPad Software) and expressed as mean ± Standard Error of the Mean (SEM) or ± Standard Deviation (SD). Statistical test used for comparing means of cells counts (HDAC2 + cells) for five degrees of nuclear localization was multiple t-tests, with significant p value determined through Holm-Sidak method as *post-hoc* test (α = 0.05) (* *p* < 0.05; ** *p* < 0.01; *** *p* < 0.001). Statistical analysis on both western blot and RT-qPCR data was performed using Student t-test, considering significance for p value < 0.05. Variance of means of three groups in AlphaLISA assay was compared using one-way ANOVA, with *p* < 0.05 considered significant (* *p* < 0.05; ** *p* < 0.01; *** *p* < 0.001).

## Results

### Identification of novel pathogenetic variant in *HDAC2* gene

By WES analysis we found a *de novo* pathogenetic variant in *HDAC2*, located on chromosome 6q21 on a patient, previously tested negative for RSTS causative known genes.

**Fig. 1:**
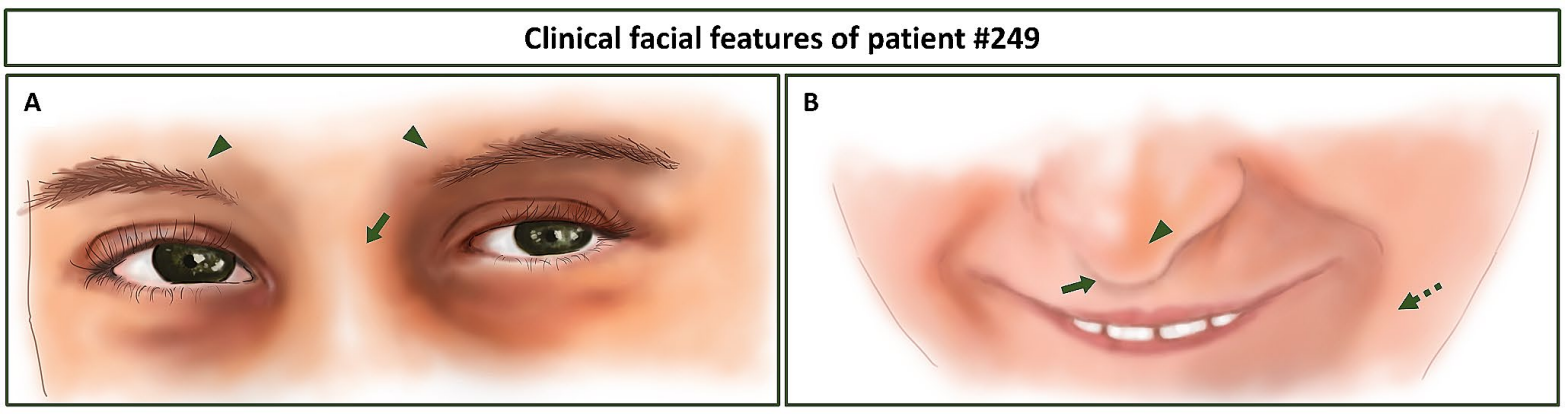
Sketch of clinical facial features of patient #249. (**A**) Eye-nose root details showing well-defined eyebrows with a mild synophrys (arrowheads) and high nasal root (arrow). (**B**) Nose-mouth details such as prominent columella (arrowhead), short philtrum (arrow) and prognathism (dashed arrow)

The patient displayed some RSTS features such as ID, growth and motor delay, peculiar dysmorphisms (synophrys, prominent columella, short philtrum, high nasal root, abnormal ears with prominent antihelix), broad halluces, speech delay, feeding problems and recurrent infections (Fig. 1; Table 2). In addition, she presented vertebral anomalies, hypoplasia of corpus callosum, hypermetropia and early puberty (Table 2).

**Table 2:** Clinical signs of patient #249 described in this work compared to typical features of RSTS and of other two *HDAC2* reported cases in literature. + = present; - = absent, +/- = mild presence, *NA* = not assessed

	RSTS	#249	HDAC2
Facial dysmorphisms			
Low anterior hairline	+	+/−	+
Long eyelashes	+	+	−
Synophrys	+/−	+	+
Ptosis	+	−	−
Downslanting palpebral fissures	+	−	−
Thick eyebrows	+/−	+	+
Hypertelorism	+	−	+
Low hanging columella	+	+	−
Convex and wide nasal bridge	+	−	−
Facial grimacing	+	−/+	−
High-arched palate	+	+	*NA*
Micrognathia	+	−	−
Low set ears	+	−	+
Strabismus	+	−	−
Flammeus nevus/angioma	+/−	−	−
IUGR	+/−	−	−
PNGR	+	+/−	+
Intellectual disability	+	+	+
Speech delay/absence	+/−	+	+
Behavioral problems	+/−	+/−	*NA*
Teeth anomalies	+	+	+
Skeletal anomalies			
Polydactyly	+	−	+
Broad thumbs	+	−	−
Angulated thumbs	+/−	−	−
Broad halluces	+	+/−	−
Clinodactyly	+	−	−
Brachydactyly	+	−	−
Microcephaly	+	−	−
Delayed bone age	+	−	*NA*
Hypotonia	+	+	+
Genitourinary anomalies	+	−	+
Heart defects	+	−	+
Brain anomalies	+	+	+
Seizures	+	−	−
Hypertrichosis	+	+	−
Keloids/naevi	+	−	−
Frequent infections	+	+	*NA*
Feeding problems	+	+	+
Gastroesophageal reflux	+	+	+
Vertebral anomalies	+	+	+
Others		precocious puberty, submucous cleft palate, pectus excavatum	

**Fig. 2:**
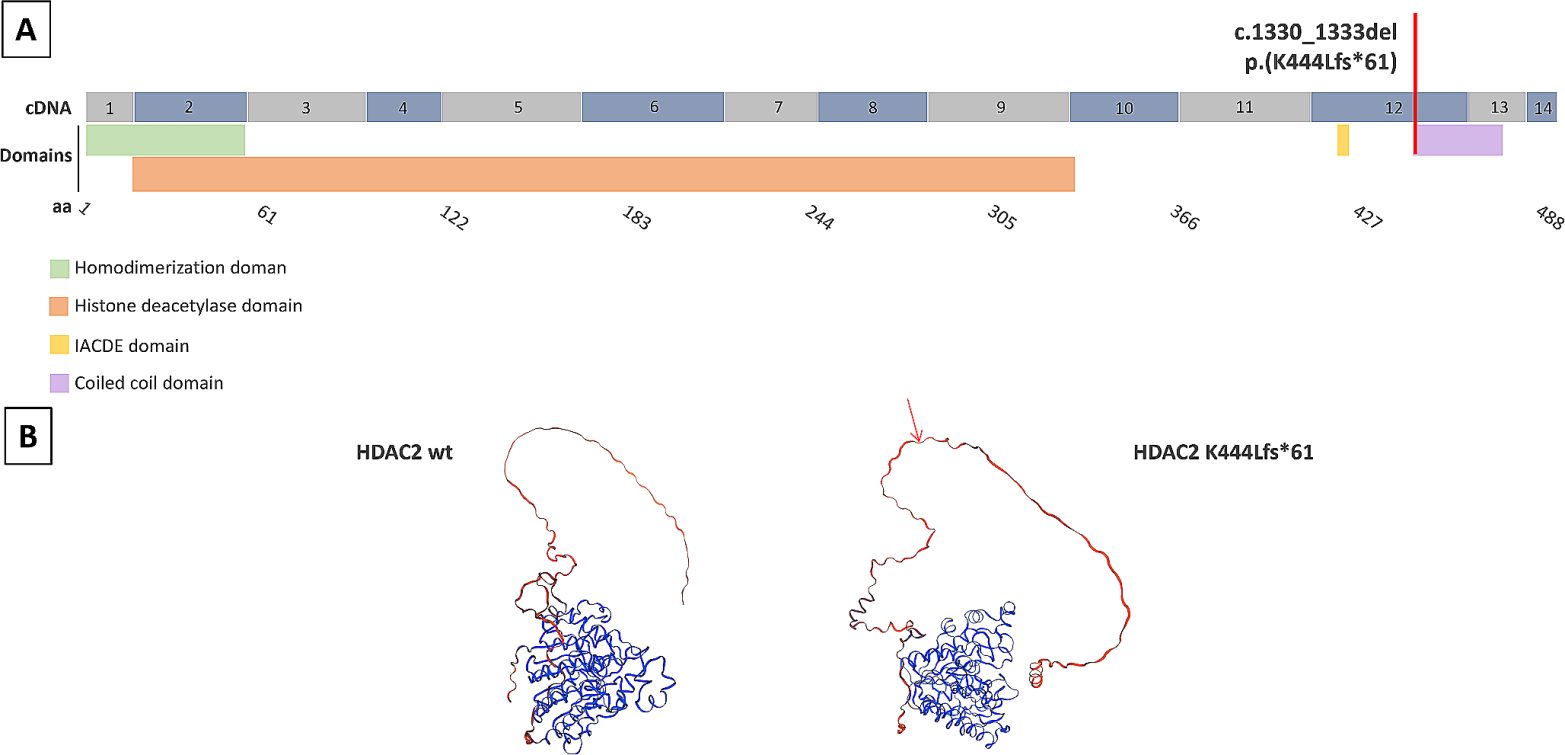
Schematic representation of *HDAC2* pathogenetic variant found in patient #249. (**A**) Localization of *HDAC2* variant (red line) at transcript (cDNA sequence with numbers referring to exons) and protein level (aa sequence) with different HDAC2 domains (homodimerization domain in green, histone deacetylase domain in orange, IACDE domain in yellow, coiled coil domain in purple). (**B**) Representation of wild type (wt) HDAC2 structure and protein with K444Lfs*61 predicted by SWISS-MODEL modelling server

From trio blood and patient #249 saliva samples we found a deletion of four nucleotides (c.1330_1333del), confirmed also on cDNA, which was predicted to cause the frameshift p.(K444Lfs*61), affecting the coiled coil domain of the protein (Fig. 2). According to ACMG guidelines, the clinical significance of the variant is likely pathogenetic (PVS1, PM2).

### Nuclear mis-localization of HDAC2 in patient LCL lymphoblastoid cell line (LCL)

**Fig. 3:**
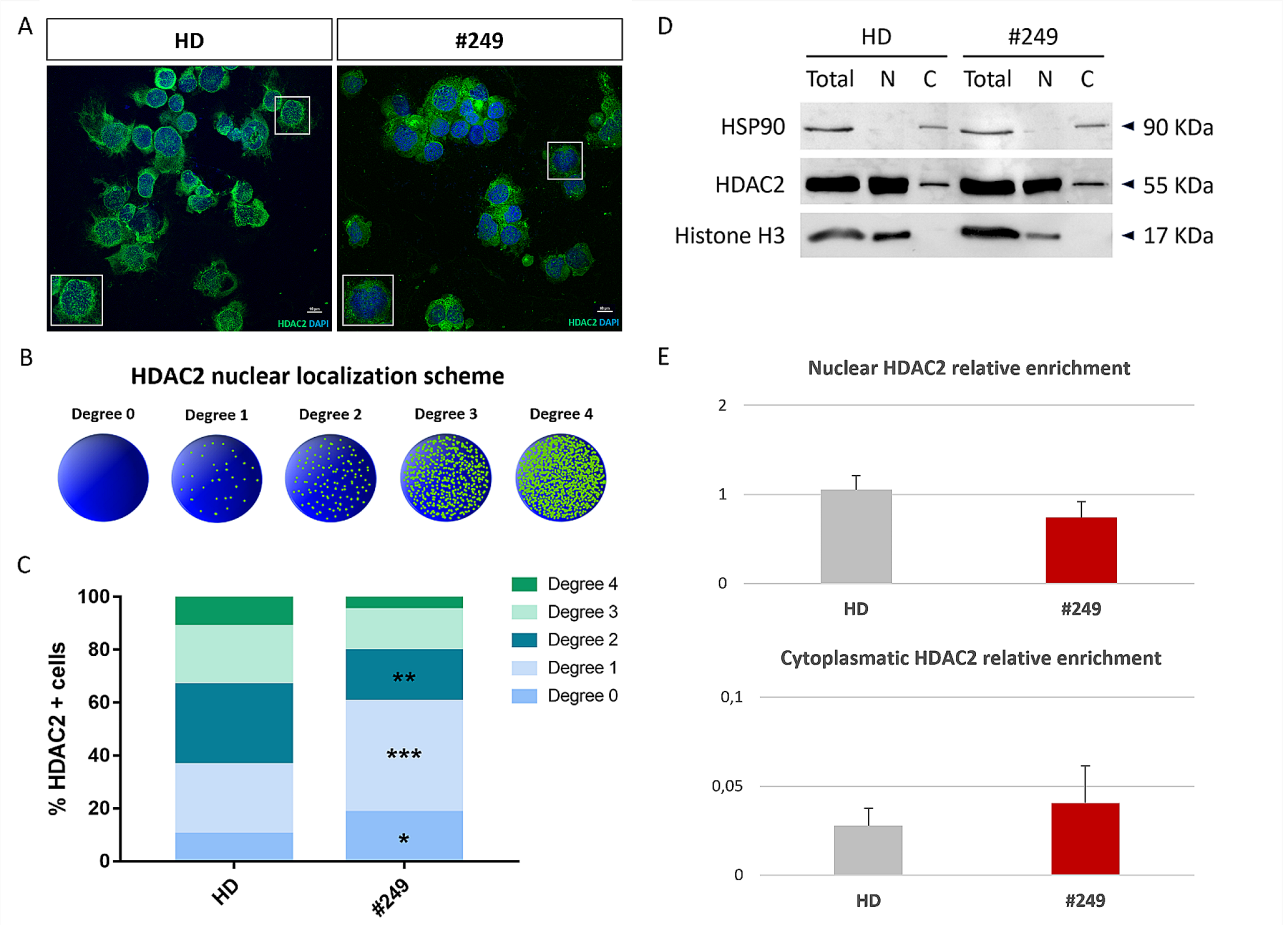
Cellular localization of HDAC2 in patient derived lymphoblastoid cell line (LCL). (**A**) 60x confocal images showing an example of HDAC2 immunocytochemistry on HD LCL and patient LCL (#249); HDAC2 is marked with green signal and nuclei with blue (DAPI); Insets show cell magnification of degree 4 (in HD box) and degree 0 (in #249 box) of HDAC2 nuclear localization. (**B**) Schematic representation of degrees set for evaluation of HDAC2 presence in nucleus, from absent (Degree 0) to most abundant (Degree 4). (**C**) Frequency of HDAC2 positive cells (% HDAC2 + cells, on Y-axis) into the five degrees of nuclear localization (in shapes from green for Degree 4 to blue for Degree 0) observed in patient (#249) and HD LCLs (on X-axis); *multiple t-test* were used as statistical method (* *p* < 0.05; ** *p* < 0.01; *** *p* < 0.001). (**D**) Western blot of HDAC2 protein (55 kDa) in control (HD) and patient (#249) LCLs lysates fractionation (total, C = cytoplasmic fraction, N = nuclear fraction) compared to cytoplasmatic marker HSP90 (90 kDa) and nuclear marker histone H3 (17 kDa). (**E**) Quantification of HDAC2 relative enrichment in HD (grey bar) and #249 (red bar) LCLs; all values are expressed as means ± SEM and data analysis was performed using *Student’s t-test* as statistical method, with p value < 0.05

Since HDAC2 localization is mainly nuclear and *HDAC2* pathogenetic variant found in patient #249 is predicted to affect the sequence of nuclear localization signal (NLS), we performed immunocytochemistry on lymphoblastoid cell lines (LCLs) and LCLs lysates fractionation to investigate HDAC2 intracellular presence (Fig. 3). Upon arbitrarily assigning five degrees of nuclear localization (from degree 0 to degree 4) as illustrated in Fig. 3B, we observed a mis-localization of HDAC2 in patient #249 compared to healthy donors (HD) LCLs (Fig. 3A and C). Degree 0–2 resulted significantly frequent in patient LCL compared to HD LCLs (*p* < 0.05 for degree 0, *p* < 0.001 for degree 1 and *p* < 0.01 for degree 2), suggesting that HDAC2 nuclear presence in #249 LCL is lower than in controls (Fig. 3C). This mis-localization was observed also performing LCLs lysates fractionation, which confirmed that HDAC2 in patient #249 has a minor nuclear enrichment compared to HD (Fig. 3D-E).

### *HDAC2* variant affects total protein abundance in patient LCL

**Fig. 4:**
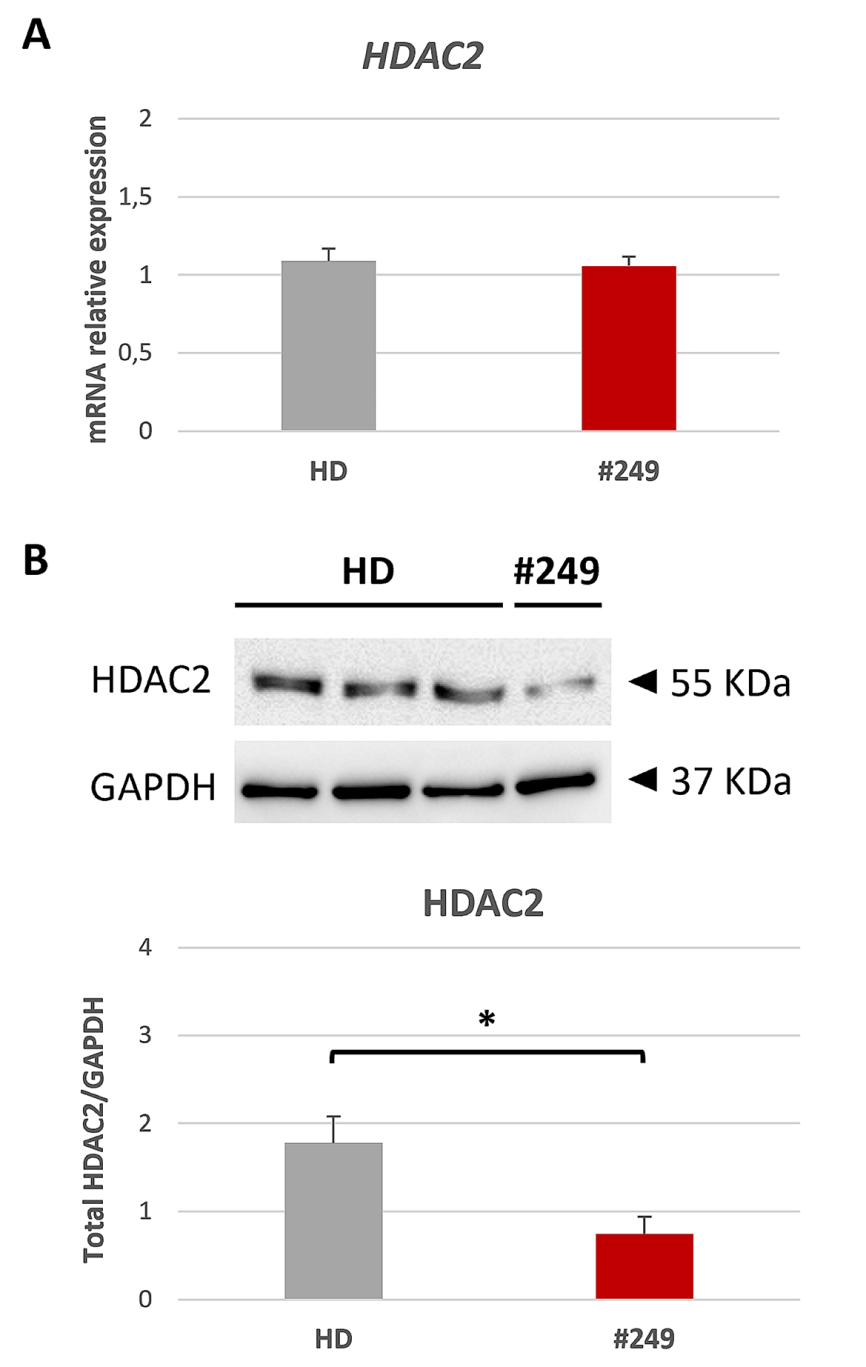
*HDAC2* relative expression and total protein abundance in patient LCL. (**A**) mRNA relative expression of *HDAC2* in five controls (HD, grey bar) and patient LCL (#249, red bar). (**B**) Western blot of HDAC2 protein (55 kDa) in three HD and #249 LCLs normalized on GAPDH (37 kDa), as represented in the quantification below. All values are expressed as means ± SEM; data analysis was performed using *Student’s t-test* as statistical method, with p value < 0.05

To understand whether the different localization of HDAC2 in #249 LCL could be caused by an altered transcription/translation of HDAC2, we investigated both *HDAC2* expression and its total protein abundance (Fig. 4). We performed RT-qPCR on samples extracted from #249 and HD LCLs but no difference in *HDAC2* relative expression among the samples was observed (Fig. 4A). Surprisingly, when we investigated HDAC2 total protein abundance in #249 compared to HD LCLs we found it significantly reduced (Fig. 4B).

### Altered acetylation pattern and p53 levels in patient LCL

**Fig. 5:**
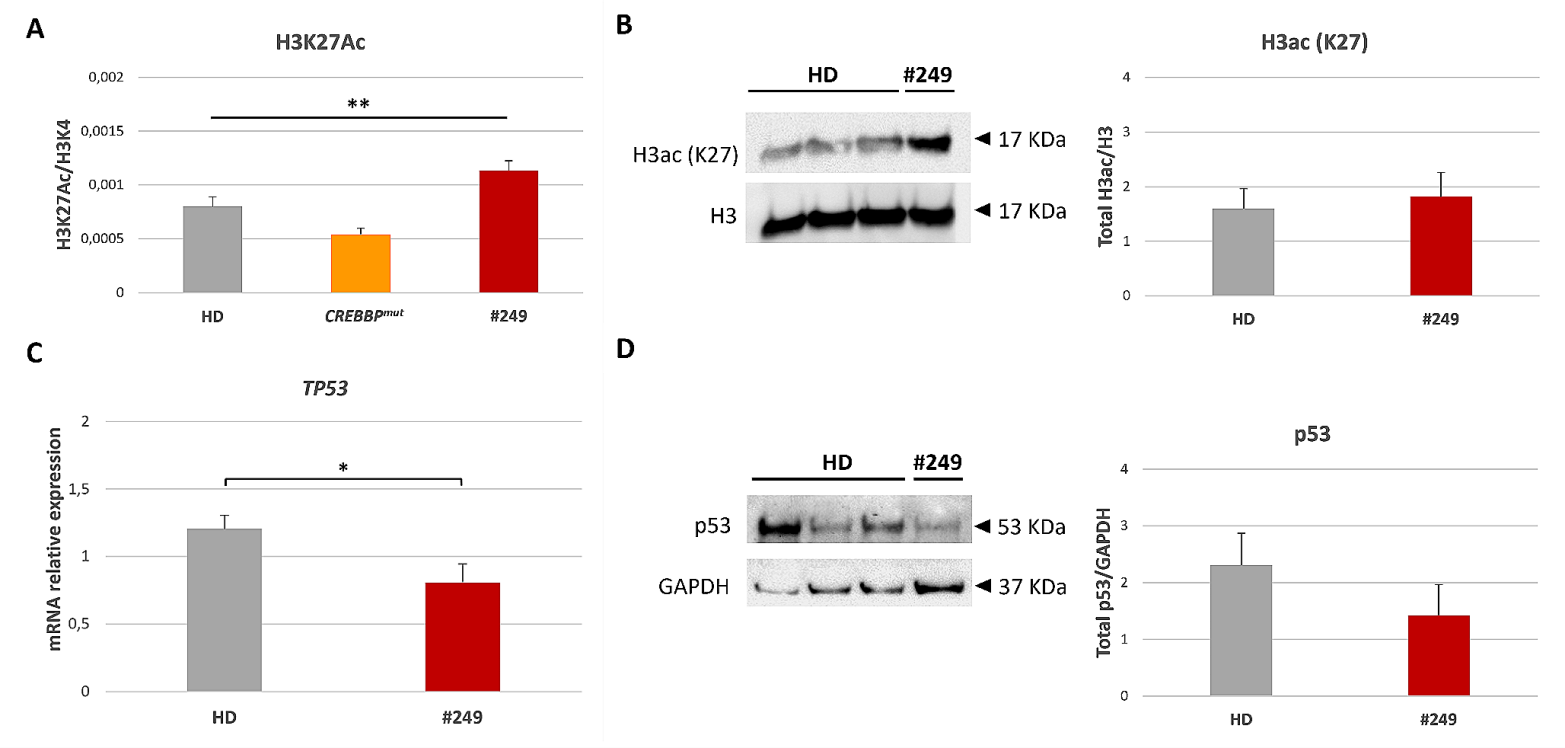
Histone acetylation and expression of HDAC2 target in patient LCL. (**A**) Acetylation of lysin 27 of histone 3 (H3K27Ac) normalized on unmodified lysin 4 of histone 3 (H3K4) (H3k27Ac/H3K4, on Y-axis) in LCLs derived from five healthy donors (HD, grey bar), three RSTS patients with mutation in *CREBBP* (*CREBBP*^*mut*^, orange bar) and patient #249 (red bar) (on X-axis), assessed by AlphaLISA assay; variance of means of histone acetylation values of the three groups was compared using *one-way ANOVA*. (**B**) Western blot of H3K27 acetylated (H3ac) protein (17 kDa) in three HD and #249 LCLs normalized on H3 (17 kDa), as represented in the quantification on the right (HD in grey, #249 in red). (**C**) mRNA relative expression of *TP53* in five controls (HD, grey bar) and patient LCL (#249, red bar). (**D**) Western blot of p53 protein (53 kDa) in three HD and #249 LCLs normalized on GAPDH (17 kDa), as represented in the quantification on the right (HD in grey, #249 in red). **B-D**) *Student’s t-test* was used as statistical method; all values are represented as means ± SEM and significance was considered for *p* < 0,05 (* *p* < 0.05; ** *p* < 0.01; *** *p* < 0.001)

To assess whether the newly identified variant in *HDAC2* affects its enzymatic activity, we performed both AlphaLISA assay and western blot on LCLs, and we investigated the expression of a known transcriptional target of HDAC2 by RT-qPCR and western blot (Fig. 5). AlphaLISA analyses allowed comparing acetylation levels relative to lysine 27 of histone H3 (H3K27Ac) normalized on unmodified lysine 4 of histone 3 (H3K4) in LCLs, in particular between #249, HD and LCLs derived from RSTS patients with mutation in the major gene *CREBBP* (*CREBBP*^*mut*^) (Fig. 5A). We found significant differences in acetylation pattern among the three groups, assessed with one-way ANOVA (*p* < 0.01) (Fig. 5A). We observed lower H3K27Ac levels in *CREBBP*^*mut*^ compared to HD as expected, and notably, higher acetylation levels in #249 compared to both *CREBBP*^*mut*^ and HD. The same trend was observed for #249 compared to HD lysates for abundance of lysine 27 of histone 3 acetylated (H3ac) normalized on histone 3 (H3) (Fig. 5B). In addition, we observed a decreased relative expression of *TP53* in #249 compared to HD LCLs, encoding for p53, a transcription factor reported to be a target of HDAC2 (Fig. 5C) and which downregulation was confirmed also at protein level (Fig. 5D).

### Patient #249 and RSTS cell lines show similar differentially expressed genes (DEGs) involving neuronal function

**Fig. 6:**
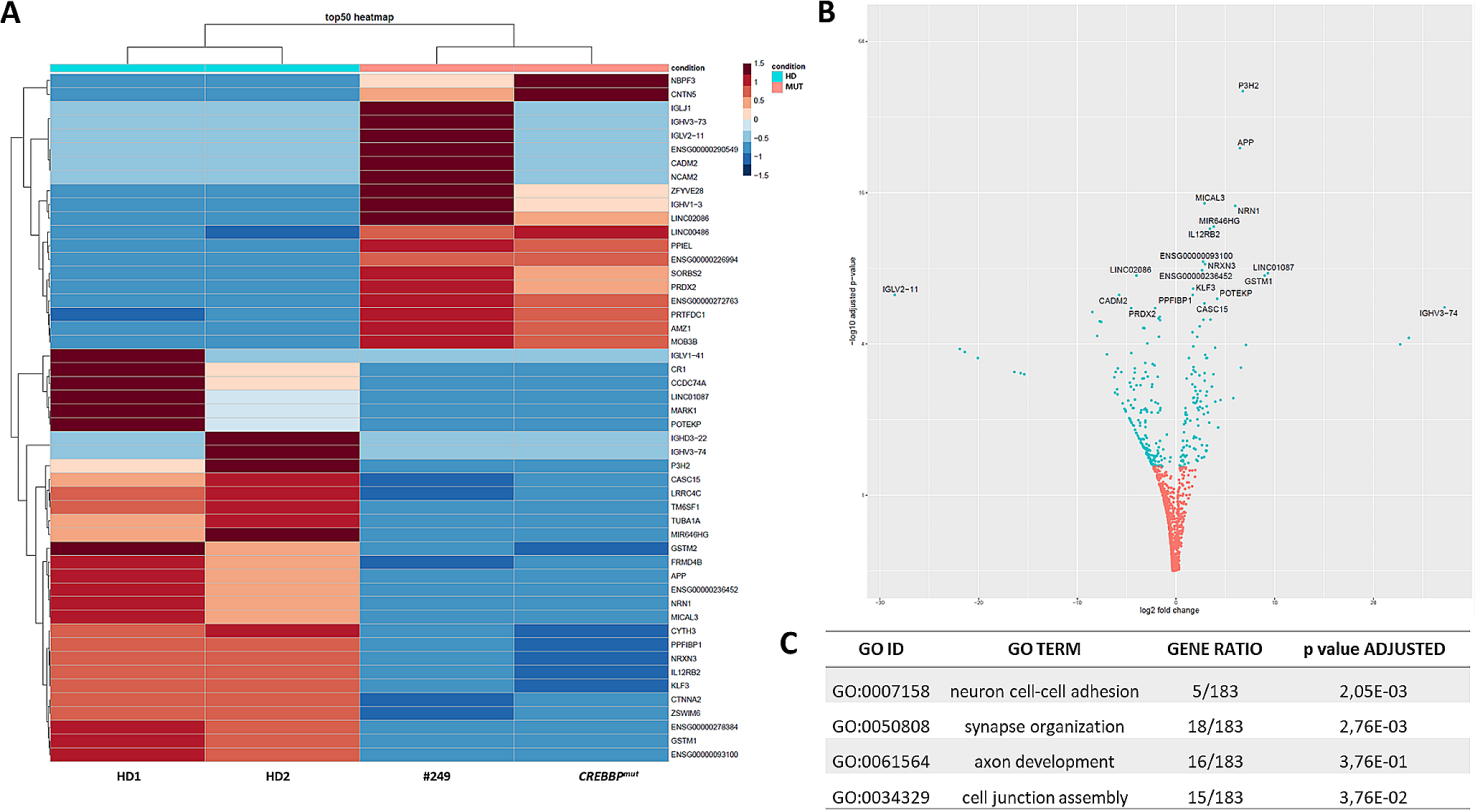
Whole-transcriptome RNA-seq analysis performed on LCLs from patient #249, RSTS and controls. (**A**) Heatmap of RNA-seq expression data showing top 50 DEGs in two controls (HD1 and HD2, also referred as HD), patient #249 and RSTS patient (*CREBBP*^*mut*^) (both referred as MUT); DEGs were selected based on nominal p value less than 0.05 and actual foldchange higher than 1.5 or lower than 0.67. (**B**) Volcano plot of DEGs between patients and controls, with log2 fold changes in gene expression on X-axis and the statistical significance (-log10 adjusted p value) on Y-axis; gene symbols of most significant DEGs (light blue) are displayed. (**C**) List of the top GO molecular functions enriched in DEGs resulted from transcriptomic analysis with respective adjusted p value

Since patient #249 was initially diagnosed as Rubinstein Taybi syndrome (RSTS) by clinical features, we investigated whether the patient’s cells shared molecular features with RSTS performing whole-transcriptome RNA-sequencing (RNA-seq) on RNA extracted from cell lines of #249, one RSTS patient (*CREBBP*^*mut*^) and two controls (HD) (Fig. 6). Interestingly, we found common differentially expressed genes (DEGs) between patients (#249 and *CREBBP*^*mut*^, both referred also as MUT) compared to controls (HD) as shown by heatmap (Fig. 6A) and volcano plot (Fig. 6B). Transcriptomic analysis revealed 302 DEGs with p-adjusted < 0.05 (top 50 genes are represented in Fig. 6A-B) and their Gene Ontology (GO) analysis showed an enrichment of genes mostly involved in neuronal function and cell junction (Fig. 6C).

## Discussion

Pathogenetic variants affecting genes of the epigenetic machinery are causative of rare disorders named chromatinopathies. Three chromatinopathies are known to be linked to HDAC genes so far (Simon et al. 2010; Deardorff et al. 2012b; Wakeling et al. 2021), and only one of them is caused by mutations in a class I HDAC gene: Cornelia de Lange syndrome 5 (CdLS5), whose affected patients are carrier of pathogenetic variants in *HDAC8* (Deardorff et al. 2012a; Kaiser et al. 2014; Parenti et al. 2016).

In this work we described a patient (#249) clinically diagnosed with Rubinstein Taybi syndrome (RSTS) who resulted negative for RSTS causative genes and for this reason underwent trio-WES. We identified an unreported *de novo* variant in *HDAC2*: c.1330_1333del, p.(K444Lfs*61), predicted to be likely pathogenetic according to ACMG guidelines. HDAC2 has a fundamental role in embryonic development, cytokine signaling, it is frequently dysregulated in cancer cells, and it affects neurological functions such as synaptic transmission and plasticity (Krämer 2009; Yamakawa et al. 2017). To date, two other *de novo* variants linked to syndromic phenotypic manifestations were reported in *HDAC2* (Martínez et al. 2017; Wagner et al. 2019). By gene panel sequencing for a syndromic intellectual disability (ID) cohort, Martinez and colleagues identified in one patient (patient #35) a *de novo* likely pathogenetic variant in *HDAC2*: c.83G > A; p.(G28D) (Martínez et al. 2017). This patient shares with the one described in this work ID, delayed speech, and developmental problems, presenting additional features such as autism spectrum disorder (ASD) behavior, hyperactivity, muscular hypotonia, stereotypies and seizures. More recently a patient clinically diagnosed as CdLS who resulted positive for a *de novo* variant in *HDAC2* by exome sequencing: c.93G > A; p.(M31I) was described (Wagner et al. 2019). Also, this patient displayed developmental and motor delay, feeding difficulties, skeletal and brain anomalies, and dysmorphisms such as synophrys, smooth philtrum, and abnormal ears. Other phenotypic features described for this patient, not present in patient #249 here reported, are cardiac anomalies, neural tube defect, cryptorchidism, hypotonia, urogenital abnormalities and other peculiar facial features. However, these two already reported variants both affects the N-terminal domain of HDAC2, the homo- and heterodimerization domain responsible for HDAC association, while the variant identified in this work is located in the coiled coil domain at HDAC2 C-terminus, important for protein-protein interactions (Ma and Schultz 2016). Although these two previously described patients have some features shared by our patient #249, a higher number of cases is needed for a deeper genotype-phenotype correlation due to the clinical heterogeneity characterizing chromatinopathies.

In addition, the novel pathogenetic variant we identified in *HDAC2* was predicted to affect nuclear localization signal. Indeed, functional analysis on lymphoblastoid cell line (LCL) derived from patient #249 showed a nuclear mis-localization of HDAC2 in #249 LCL compared to controls (HD), confirmed by immunocytochemistry and lysates fractionation. When we investigated HDAC2 expression we found it altered only in terms of total protein abundance in #249 LCLs compared to HD, while levels of *HDAC2* transcripts resulted unaffected. Thus, we investigated downstream epigenetic effects of HDAC2 and, interestingly, we observed an altered acetylation pattern of H3K27Ac in #249 LCL compared to *CREBBP*^*mut*^ and HD LCLs. Moreover, when we assessed both gene and protein expression of the transcription factor *TP53*, coding for the known target p53, we found it downregulated in #249 compared to HD LCLs. Thus, the frameshift mutation identified in patient #249 might prevent HDAC2 repressive action in the nucleus, resulting in an imbalance in histone acetylation (i.e. H3K27Ac) and causing an altered epigenetic function with effects on gene regulation. Indeed, we observed a downregulation of p53 probably due to the defective presence of both total and nuclear HDAC2, as it was demonstrated that HDAC1 and HDAC2 positively regulates p53 expression (Stojanovic et al. 2017). Since our patient #249 had an initial clinical diagnosis of RSTS we deepened genotype-phenotype correlation performing whole RNA-seq on #249, *CREBBP*^*mut*^ and HD LCLs. Interestingly, we found shared DEGs in #249 and RSTS line compared to HD, mostly involving neuronal function (i.e. neuronal cell adhesion, synapse organization and axon development), suggesting the existence of common dysregulated molecular pathway for these two conditions (Ng et al. 2023).

In conclusion, for the described RSTS patient without a molecular diagnosis, a trio-WES approach led to the identification of a novel variant in a gene not previously associated to chromatinopathies. Although collecting new patients is required for suitable genotype-phenotype correlation, the other two reported cases with *HDAC2* mutations affecting the N-terminal domain and with overlapping phenotype with the patient here described, could be a starting point for ascribing this condition to a chromatinopathy. In addition, molecular investigation of this *HDAC2* variant could represent preliminary findings for disclosing pathogenetic mechanisms underlying this neurodevelopmental disorder affecting the epigenetic machinery.

## Data Availability

FASTQ files are available in the Biostudies-ArrayExpress database (https://www.ebi.ac.uk/biostudies/ArrayExpress/studies) under accession number E-MTAB-14042.
